# Development and Characterization of an Orodispersible Film for Vitamin D3 Supplementation

**DOI:** 10.3390/molecules25245851

**Published:** 2020-12-11

**Authors:** Irma Elisa Cupone, Eleonora Dellera, Fabio Marra, Andrea Maria Giori

**Affiliations:** IBSA Farmaceutici Italia, 26900 Lodi, Italy; irma.cupone@ibsa.it (I.E.C.); eleonora.dellera@ibsa.it (E.D.); fabio.marra@ibsa.it (F.M.)

**Keywords:** vitamin D3, immune system, food supplement, orodispersible film, innovative dosage form, mechanical properties

## Abstract

Vitamin D plays a crucial and very well-known role in regulation of calcium homeostasis and bone metabolism and mineralization. However, a huge and more recent body of evidence supports the positive influence of vitamin D on the regulation of immune response, ranging from protection against respiratory tract infections to prevention and management of asthma. Nevertheless, vitamin D deficiency is a very common condition and there is an increasing need for suitable products for proper supplementation, allowing good compliance also in specific populations. Orally disintegrating tablets (ODT) were first developed to overcome the difficulty experienced by pediatric and geriatric patients of swallowing traditional oral dosage forms and, recently, orodispersible films (ODF) are gaining popularity as novel dosage form for assuming active pharmaceutical ingredients, vitamins, and ingredients for food supplements. This study describes a 2000 IU Vitamin D3 ODF for daily intake, consisting of hydrophilic polymers and suitable excipients, manufactured by film-casting process. Elongation-at-break (E%), Young’s modulus (Y), and tensile strength (TS) were investigated using a dynamometer. Chemical stability was evaluated assaying the vitamin D3 in the films stored at different environmental conditions. In addition, in vitro disintegration and dissolution studies were performed. Correlation existed between the mechanical properties of the film and the residual water, acting as plasticizer. The stability study showed that vitamin D3 assay was ≥90% also after 3 months at 40 °C. The film disintegrated in less than 1 min and the vitamin D3 released was ≥75% after 15 min. An ODF with suitable properties can be manufactured and used as innovative dosage form for vitamin D3 food supplements.

## 1. Introduction

Vitamin D has for a long time been known for its activity carried out in bone health, by promoting calcium absorption in the gut and maintenance of serum calcium and phosphate concentration [1]. As vitamin D is a central regulator of calcium homeostasis and bone metabolism, vitamin D deficiency in adults results in poorly mineralized skeletal matrix, a symptom known as osteomalacia, and is known to cause osteoporosis and muscle weakness, increasing the risk for fractures due to falls, especially in the elderly [2].

More recently, the role of vitamin D as regulator of the immune system was investigated. Vitamin D acts as an important stimulant for innate immunity and supports the adaptive immune system [3]. Vitamin D3 has been recognized as an important mediator of innate immune responses, enhancing the antimicrobial activity of immune cell, such as monocytes and macrophages [2].

Moreover, vitamin D can increase T regulatory lymphocytes, that are the principal defense against uncontrolled inflammation and against viral infection in general. Low levels of vitamin D are also associated with an increase in inflammatory cytokines [4].

Vitamin D can reduce risk of infections through several mechanisms, such as the induction of cathelicidins and defensins that can lower viral replication rates.

Recent data reported the direct antiviral effects of vitamin D, which hinders viral replication. Vitamin D can also be effective in an anti-inflammatory and immunomodulatory way, reducing concentrations of pro-inflammatory cytokines, responsible for the damage to the lining of the lungs, which leads to pneumonia, as well as increasing concentrations of anti-inflammatory cytokines. Vitamin D could be an effective means of preventing respiratory tract infection [5]. Analysis indicated that daily or weekly intake of Vitamin D showed protective effects against acute respiratory tract infection especially in person with vitamin D deficiency. In clinical studies, low levels of serum vitamin D were associated with acute respiratory tract infections including epidemic influenza. Some recent reviews hypothesized that vitamin D insufficiency may compromise respiratory immune function, increasing the risk of COVID-19 severity and mortality [6].

Vitamin D deficiency (serum level <50 nmol/L or 20 ng/mL) is present in 24% of US population, 37% of Canada population, and 40% of Europeans. In addition, even more severe deficiency defined as serum level <30 nmol/L or 12 ng/mL) is reported in 5.9% in US, 7.4% in Canada, and 13% in Europe. Vitamin D deficiency may vary by age, with lower levels in childhood and the elderly, and also ethnicity in different regions [7].

In humans, vitamin D can be obtained from two distinct sources, either from diet or by endogenous production, whereby solar UV-B irradiates 7-dehydrocholesterol present in the skin to generate cholecalciferol (also named vitamin D3), which is subsequently activated in the liver and kidney [8].

Since sunlight exposure and dietary intake alone is usually insufficient in most individuals to maintain optimal vitamin D status, there is an increasing need of suitable products for proper supplementation, allowing good compliance also in specific populations at risk, such as the elderly and children.

Currently, there is no international consensus on the optimal level for vitamin D supplementation. Recommendations differ in many countries, and range from 400 to 2000 IU daily (10–50 μg). The European Food and Safety Authority recommend staying below 4000 IU/day (100 μg). Most countries have prudently set the safe upper level at 50 μg daily (2000 IU) for adults [7].

The aim of this study is to develop a 2000 IU Vitamin D3 orodispersible film (ODF) for daily supplementation that is easy to use and characterized by an improved compliance when compared to the existing oral dosage forms.

ODF is a novel dosage form for taking active pharmaceutical ingredients, vitamins and ingredients for food supplements.

According to the European Pharmacopoeia (Ph. Eur.), ODFs are defined as “single or multilayered sheets of suitable materials, to be placed in the mouth where they disperse rapidly”. ODFs require only a small amount of saliva to dissolve within a few minutes and water is not necessary for administration. The advantage is the improvement of acceptance and patient compliance with no risk of choking, associated with better safety and efficacy in comparison with conventional dosage forms [9]. ODFs allow overcoming the difficulty in swallowing conventional oral dosage forms among pediatric, geriatric, and psychiatric patients with dysphagia. Their convenience, superior dosing accuracy, and rapid onset of action contribute to strong patient preference over conventional solid dosage forms across a wide range of patient groups [10].

Moreover, orodispersible films have advantages over orally disintegrating tablet formulations, which may require more complicated and expensive manufacturing processes, have issues of hardness and friability during manufacturing, storage, handling, and administration.

Orodispersible films are thin and flexible, can be manufactured in a wide range of sizes and shapes, and are easily transported and stored.

Essential excipients of ODFs are polymers, which are the backbone of film formulations (40–50% of film mass) and plasticizers, usually added up to 20% of dry polymer weight. Film forming polymers have to disintegrate or dissolve rapidly in contact with saliva, have to guarantee suitable mechanical properties, and avoid stickiness. Plasticizers reduce glass transition temperature of the polymer, reduce brittleness, improve mechanical properties of films, and improve flexibility. Other components can be: taste masking agents and sweeteners that mask bitter and unpleasant taste that characterized the majority of drug substances; surfactants that act as solubilizing, dispersing, and wetting agents and improve coating step during manufacturing of ODFs by casting methods.

Due to their moderate size and thickness, ODFs can typically be loaded only with limited active substance amounts per unit volume and surface area. Therefore, oral films are not generally suitable for treatments requiring high dosages (i.e., >100–150 mg), although up to 50% drug loading in an ODF has been reported [11]. Drug dose can be modulated modifying the size and the thickness of the film and the high drug loading capacity of ODF allows the reduction of excipients intake.

ODFs can be manufactured by different methods: solvent casting, hot melt extrusion, semisolid casting method, rolling method, or electrospinning [12].

Ideal ODFs should exhibit adequate elasticity, flexibility, softness, mechanical properties to facilitate their production, packaging, and application, good stability, short disintegration time, and pleasant taste.

The flexibility and toughness of the film affect manufacturing process of films such as cutting, film formation, and packaging. Oral film should be flexible to be handled without failure and at the same time should exhibit a tensile strength that guarantees a suitable toughness to allow films to be self-supporting. Elongation at break should be low to avoid deformation of films during manufacturing process.

Taste is an important factor in the development of ODF to ensure patient acceptability and compliance and is one of the prime factors determining market penetration and commercial success. Palatability and pleasant taste are necessary for fast dissolving films, and various techniques are available to mask drug taste. Flavors and sweeteners can be added to the formulation, generally in association with other taste-masking components. Mouthfeel and texture are also considered key characteristics, potentially affecting the acceptability of ODFs, particularly with regards to the presence of residual particles following disintegration.

This work describes the development of a new vitamin D3 orodispersible film and the study to scale up the product to the manufacturing plant. The new vitamin D3 ODF was conceived as food supplement, consisting of hydrophilic polymers and suitable excipients, and manufactured by film-casting process. Critical quality attributes—such as vitamin D assay, mechanical properties, moisture content, disintegration time, dissolution profile, and stability—were investigated.

## 2. Results and Discussion

### 2.1. Selection of Components and Formulation Development

The main ingredient of Vitamin D3 oral film is maltodextrin. We started the development of the product considering the patent EP1689347 “Self supporting film for pharmaceutical and food use” and the patent WO2014/049548 “Orodispersible films having quick dissolution times for therapeutic and food use” [13,14], that required the use of maltodextrin as the only film forming ingredient. This polymer provides advantages in terms of palatability, physical properties, and stability. This ingredient was successfully used previously to design a pharmaceutical oral film formulation containing Sildenafil Citrate. In vivo study showed that such formulation was safe, efficacious, and bioequivalent to the conventional branded film-coated tablets [10]. Starting from this positive experience in the pharmaceutical field, we decided to apply for the first time the same technology to a food supplement. Maltodextrins (MDXs) are natural, safe, and cheap polymers widely used both in pharmaceutical and food applications. They are water soluble film forming biopolymers and, according to the patents, an amount of maltodextrins ranging between 40% and 80% allows to obtain a rapidly dissolving self-supporting and edible film. MDXs are a non-sweet, nutritive saccharide mixture of polymers that consists of D-glucose units, with a dextrose equivalent (DE) less than 20. MDXs are obtained by partial hydrolysis of a food grade starch with suitable acids and/or enzymes. DE is defined as the measure of the total reducing power of all sugars present in the hydrolysate material relative to glucose as 100 and expressed on a dry weight basis. DE is an indicator of the degree of depolymerization of starch: the DE value of the MDXs increases as the average molecular weight decreases. Several physical and functional characteristics are affected by the DE value. The solubility, sweetness, and hygroscopicity increase with increasing DE, whereas the viscosity, the anti-crystallizing power, and the freezing temperature decrease as the DE increases. DE value of 6 was selected, as it guarantees film formation ability and suitable tensile properties.

To improve the mechanical properties of the orodispersible film, the addition of plasticizers and surfactants is mandatory.

Glycerol and mannitol are below to the chemical group of polyols and are used as plasticizers. Plasticizers improve the flexibility and reduce the brittleness of orodispersible films, reducing the polymer glass transition temperature. They help to overcome brittleness after the step in the drying tunnel and the following punching and pouching in the primary packaging. In addition, residual water also acts as a plasticizer.

Glyceryl monolinoleate and polysorbate 80 were the surfactants chosen. Glyceryl monolinoleate consists of mono-, di-, and triglycerides of mainly linoleic (C18:2) and oleic (C18:1) acids, is a liquid water insoluble surfactant with an HLB = 1. Polysorbate 80 is a non-ionic surfactant, it is a viscous water-soluble yellow liquid with an HLB = 15. In the formulation of vitamin D3 orodispersible film a mixture of glyceryl monolinoleate: Polysorbate 80, 75:25% *w*/*w*, was used and the calculated value of HLB is 4.5.

An equilibrated HLB was selected to improve the spreadability of the water-based mass on the silicone/PET release liner during coating step and to avoid the foaming during the mixing.

Surfactants have also an important role in dissolution of Vitamin D3, acting as dissolution enahncers. To improve the homogeneity of the bulk, it is recommended to solubilize the active substance before mixing. However, vitamin D3 is a very lipophilic substance, insoluble in the water used as solvent for MDXs and other hydrophilic excipients. To overcome these problems, extra virgin olive oil was used as solubilizing agent for vitamin D3. Vitamin D3 is then added to the mixture as oil solution and the surfactants comprised in the formula allowed the complete and homogeneous dispersion of vitamin D3 solution in the aqueous mass. Therefore, even if vitamin D3 is a poorly water soluble substance, the use of organic solvent to obtain a homogeneous mixture is not necessary [15], with advantages in terms of safety of both manufacturing process and finished product.

Copovidone and alginate were also introduced as film forming agents able to improve the mechanical properties of the films. Copovidone was introduced in the film formulation according to the patent WO 2014/049548: this patent teaches that it is possible to avoid hardening of the films, based on maltodextrins and plasticizer, adding a homopolymer or copolymer of vinyl acetate to the composition.

Alginate acts also as a thickener, and was add to the formulation mainly for this role. Alginate is a hydrocolloid, explicitly excluded by both patents of reference on maltodextrins platform. The addition of alginate improved the physical stability of the mixture (data not shown). That is the main difference between the formula described in this paper and the ones claimed by the cited patents.

The low concentration of vitamin D3 in the film does not affect the pleasantness, but, in order to improve the organoleptic characteristics and to make the product more agreeable, low amounts of sweetener and flavor were added to the formula. Sucralose was selected as sweetener while orange, apricot, and peach flavors were tested. Among them, after an expert panel evaluation, orange flavor was considered the most suitable for this kind of formulation.

Titanium dioxide is a white pigment used to improve film aspect and was combined with yellow iron oxide and red iron oxide to obtain a light orange colored film.

Vitamin D3 is sensitive to air, heat, humidity, and light and accordingly ascorbic acid and DL-alfa tocopherol are introduced as antioxidant agent to improve stability of Vitamin D3. Vitamin C and Vitamin E acted synergically in combination [16,17]. The two vitamins were added in 1:1 weight ratio.

An overage of Vitamin D3 was loaded in the product (+20%) in compliance with the European regulation on food supplement. Vitamin D3 film with labeled strength of 2000 IU, corresponding to 50 μg, contains a nutrient level of 60 μg. Such amount of Vitamin D3 was loaded on a film of 6 cm^2^, with a concentration of active ingredient of 0.06% *w/w*.

Final composition and function of each ingredient of developed vitamin D3 orodispersible film are reported in Table 1.

### 2.2. Manufacturing Process Development

The manufacturing of Vitamin D3 orodispersible films was performed according to an IBSA standard process developed and validated for the manufacturing of other orodispersible film already on the market.

Zhang et al. [18] has already described an oral fast dissolving film containing Vitamin D3 obtained by microemulsifying; these films were prepared in laboratory on a bench and no evidence of feasibility of industrial manufacturing was provided.

Manufacturing process of vitamin D3 ODF was previously studied in laboratory, then the process was scaled up to the industrial plant. Water content and viscosity of the mass were identified as two important quality attributes of the mass in the scale up of the process and were investigated during laboratory studies in order to obtain a mass suitable for industrial process. Previous studies on orodispersible films demonstrated that the suitable viscosity value has to be in the range 2000–5000 cP. Vitamin D3 mass was formulated to have a viscosity of about 3500 cP: this value is suitable for industrial coating process and is the result of a mixture containing the 39.0% of water.

Mass manufacturing process was developed in laboratory using 5 L mixer equipped with anchor and high disk speed: order of addition of components and time of mixing necessary to obtain a homogeneous mixture were identified as critical process parameters

Industrial mixture was prepared in a 50 L stainless steel mixer equipped with anchor and high disk speed and critical process parameters were set on the basis of lab scale.

The mixture, homogeneous and without bubble and clumps, was spread on a support, a siliconized PET sheet moving along the oven, that at the end is coiled up onto a jumbo roll (Figure 4a). According to our knowledge, temperature of ovens was set between 90–110 °C and coating speed at 0.5 m/min. These conditions were suitable to remove the excess of water and the industrial continuous process allowed the production of a film with a homogeneous and smooth surface, a weight of 167 g/m^2^, and a thickness in the range 125 μm ± 5% (Table 2). The size and the strengths of the orodispersible film are defined by the consecutive processes of slitting (Figure 4b) and formation. In the first step, jumbo rolls were cut in 7 reels of 30 ± 1 mm. The 30 mm reels are cut by a single rotary die blade with a cutting pitch of 20 mm originating the 2000 IU orodispersible film (20 × 30 mm).

During the film formation, the support is removed.

Each orodispersible film was individually inserted between two packaging foils that were sealed by two sealing stations. A rotating knife cuts the single packaging unit after sealing. The variable data are printed on the sachet on the line. Secondary packaging is the last step of manufacturing process.

The industrial manufacturing process allows to obtain the following theoretical quantities of intermediate and films:

- Wet mixture: 50 Kg
- Coated laminate: 200 m^2^
- Reel: about 6000 linear meter
- Films: about 300,000 films of 2000 IU (3 × 2 cm)

**Figure 1:**
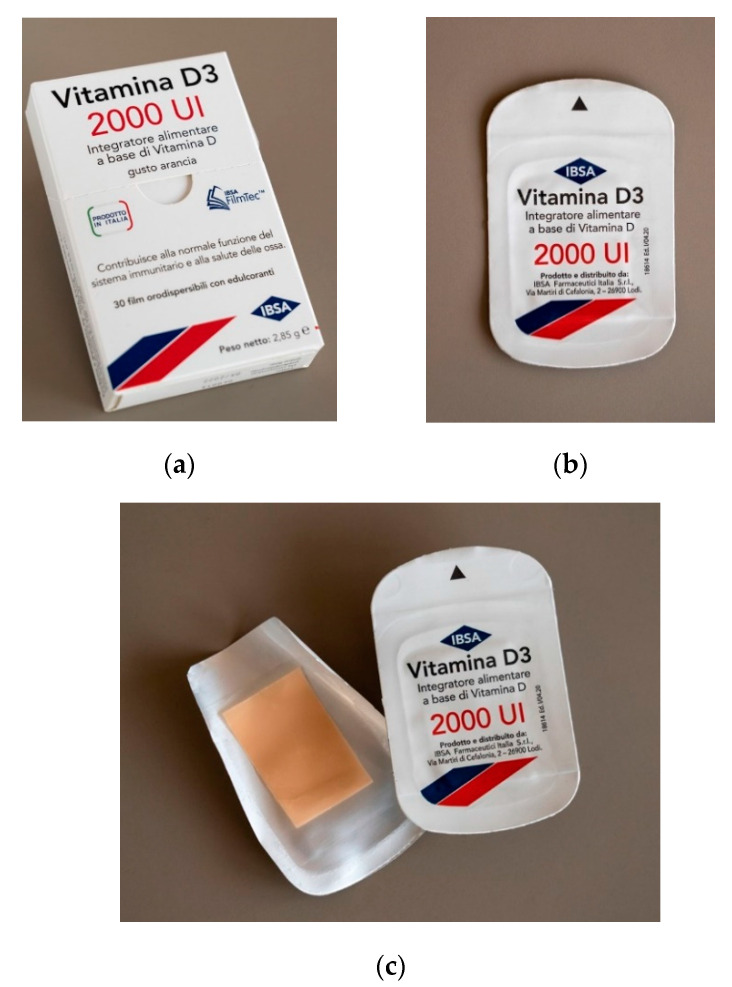
Vitamin D3 orodispersible films: secondary packaging (**a**), primary packaging (**b**), and an oral film in the sachet (**c**).

Figure 1 shows Vitamin D3 orodispersible films obtained on industrial scale and manufacturing for sale.

### 2.3. Characterization of Vitamin D3 Orodispersible Films

The critical issues in the development the formulation of an orodispersible film are represented by the time of disintegration in the oral cavity, the tensile properties required for packaging and handling procedures, and the taste of the ingredients.

At the end of the development, the formulation selected was suitable for the industrial manufacturing process. The films were characterized and results are described below.

A thin and flexible light orange film was obtained, the color was homogeneous and it was characterized by a pleasant odor and taste of orange fruit. Orodispersible films containing 2000 IU of vitamin D3 measured 20 × 30 mm and weighed 100.0 mg. Average vitamin D3 content is 60.3 µg/film, corresponding to 120.7% of label content.

**Figure 2:**
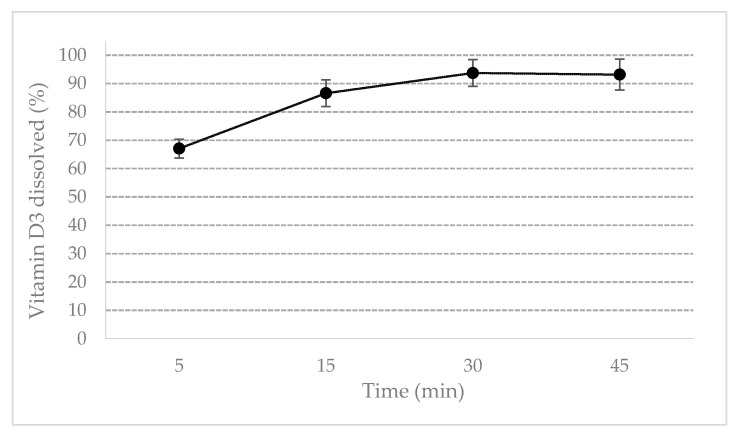
Dissolution profile of vitamin D3 ODF.

ODF disintegrated within 1 min therefore making vitamin D3 immediately available to dissolve. As shown in Figure 2, vitamin D3 dissolved completely (>90%) within 30 min, but already after 15 min the dissolved amount of vitamin D3 was greater than 85%, satisfying the requirements for the immediate release dosage forms. Vitamin D3 can be promptly bioavailable to be absorbed.

### 2.4. Evaluation of Mechanical Properties

The flexibility and stiffness of the film could affect cutting, film formation and packaging during the manufacturing process.

To facilitate the handling, oral film should be flexible and exhibit a tensile strength (TS) that guarantees a suitable stiffness. Elongation at break (E%) should be low to avoid deformation of films during manufacturing process.

The main responsible of the mechanical properties of the film is the film forming polymers, while the amount of plasticizers modulated the mechanical properties. Glycerol is the main plasticizer, but also the residual water, the amount of water remaining after the drying, acted as a plasticizer in the formulation. We studied the relation between the water content and the mechanical properties of the film.

Table 2 reports the water content and the tensile properties of ten different vitamin D ODF prepared in laboratory modifying slightly the drying time. Water content ranged between 8.1 and 10.6%. All tested films were flexible, handy, and not brittle. A correlation between tensile properties and water content was proved: TS decreased and E% increased proportionally to water content.

**Figure 3:**
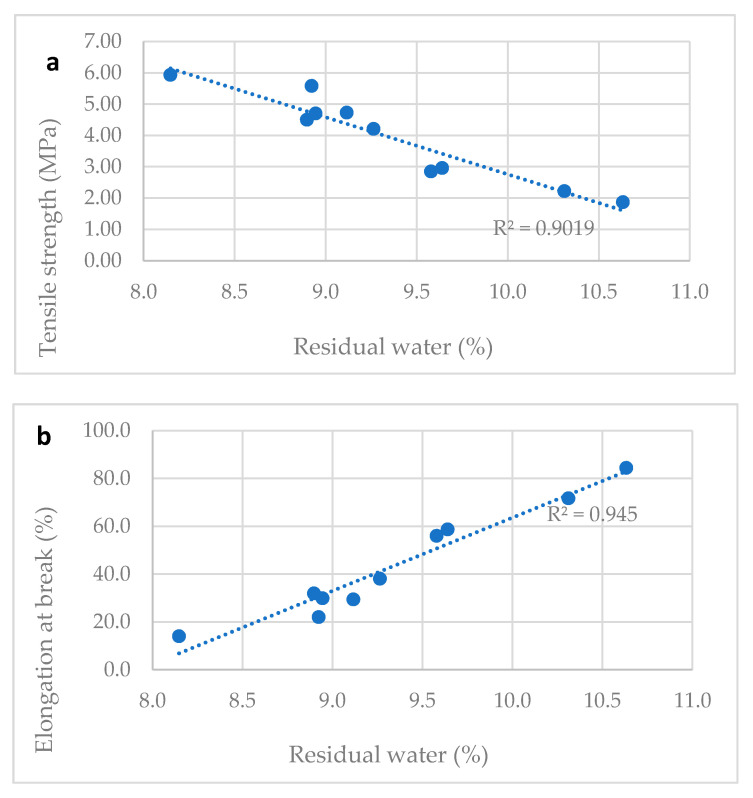

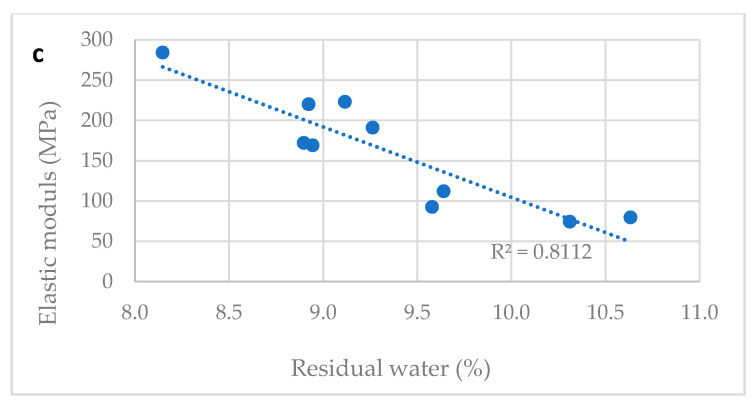
Correlation between water content and mechanical properties of films. (**a**) Tensile strength and water content correlation; (**b**) elongation at break and water content correlation; (**c**) elastic modulus and water content correlation.

The Figure 3a–c show correlation between tensile properties of vitamin D3 orodispersible films and water content. Linear correlations between elongation at break and water content (R2 = 0.945) and tensile strength and water content (R2 = 0.902) and elastic modulus and water content (R2 = 0.811) can be determined. The water content is a parameter that can predict the tensile properties of film: a residual amount of water ranging between the 8.0–11.0%, was necessary to provide the film with the suitable plasticity and tensile strength. Thus, the drying step of the manufacturing process was tuned to comply with this specification, allowing the handling of the dried film during slitting, film formation, and packaging steps on industrial plants.

### 2.5. Stability Data of Vitamin D3 Orodispersible Films

Stability data of industrial batch of film kept at the different ICH conditions are reported in Table 3. Several parameters were monitored: residual water, vitamin D3 assay, the weight, the water activity, and microbiology. The residual water remained in the range 8.0–11.0%, that guaranteed suitable mechanical properties as demonstrated above, throughout the examined period.

The weight of the films remained in the range 90–110% and weighs of films at three months are not significantly different from T0. Stability specification for vitamin D3 assay was set to 80.0 ÷ 135.0% of theoretical amount in accordance with European regulations for food supplements. Vitamin D3 assay did not decrease after three months: it remained 122.1% of the claimed value also after three months at 40 °C. Vitamin C and vitamin E protected vitamin D3 from degradation.

Water activity measures the vapor pressure of free water, a key parameter in the quality control of moisture sensitive products or materials. Free water is the water available to chemical reactions and to support microbial growth in pharmaceutical and food products, that can reduce shelf life. Water activity value 0.600 is recognized to be the critical value acceptable to prevent microbial contamination. The results of the analysis of water activity on the samples stored at 25 °C ± 2 °C/60% RH ± 5% RH and 40 °C ± 2 °C/75% RH ± 5% RH were below 0.600 and not significantly different from T0, granting the unlikelihood of a microbiological contamination during the shelf life, as confirmed by microbiological data, reported in the Table 3. Water activity is a useful and fast method to establish microbial stability.

## 3. Material and Methods

### 3.1. Materials

Vitamin D3 (DSM, Village Neuf, France), maltodextrin and mannitol (Roquette, Lestrem, France), glycerol, (Oleon, Ertvelde, Belgium), orange flavor (Sensient, Milwaukee, WI, USA), extra virgin olive oil (Balestrini, Milano, Italy), copovidone and polysorbate 80 (Eigenmann & Veronelli, Rho, Italy), vitamin C (ACEF, Fiorenzuola d’Arda, Italy), vitamin E (BASF, Ludwigshafen, Germany), glycerol monolinoleate (Gattefossè, Saint-Priest, France), titanium dioxide (Brenntag, Essen, Germany), sucralose (Sunvision Sweet, Xintai City, China), yellow iron oxide and red iron oxide (Aromata Group, Bresso, Italy), and alginate (FMC Corporation, Philadelphia, PA, USA).

All ingredients used are food grade in accordance with European Regulation.

All the solvents for the analysis were of analytical grade, unless specified, purchased from VWR (VWR, Milano, Italy).

Cholecalciferol reference standard is supplied by EDQM (European Pharmacopoeia Chemical Reference Substances).

### 3.2. Methods

#### 3.2.1. Film Preparation

**Figure 4:**
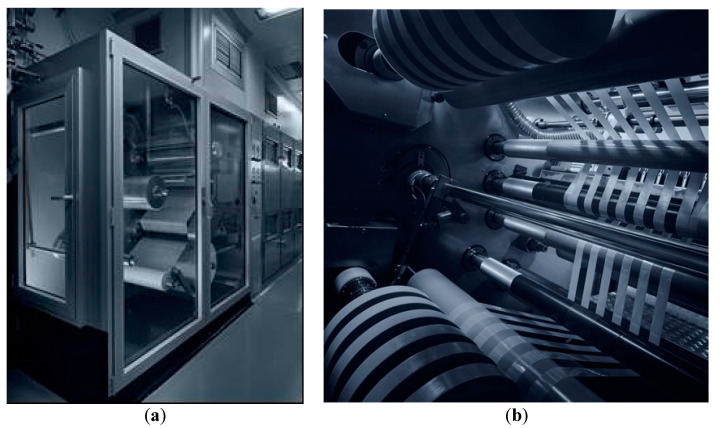
Coating (**a**) and slitting (**b**) process.

Vitamin D3 orodispersible film was manufactured according to the casting method consisting of different steps. Generally, at first, a water-based mixture was prepared, under the control of the temperature and the stirring speed of the mixer. Afterwards, the mixture was spread on the intermediate liner and dried in a drying tunnel, controlling temperature, air circulation, and coating speed. The roll of dry mass was submitted to the slitting process, to cut the roll in the reels with the first dimension of the final surface of the films. In the last step, the films are punched, pouched, and sealed in suitable single-dose sachets, using pouch-forming and sealing machine. Figure 4 shows pictures of coating and slitting manufacturing steps.

**Figure 5:**
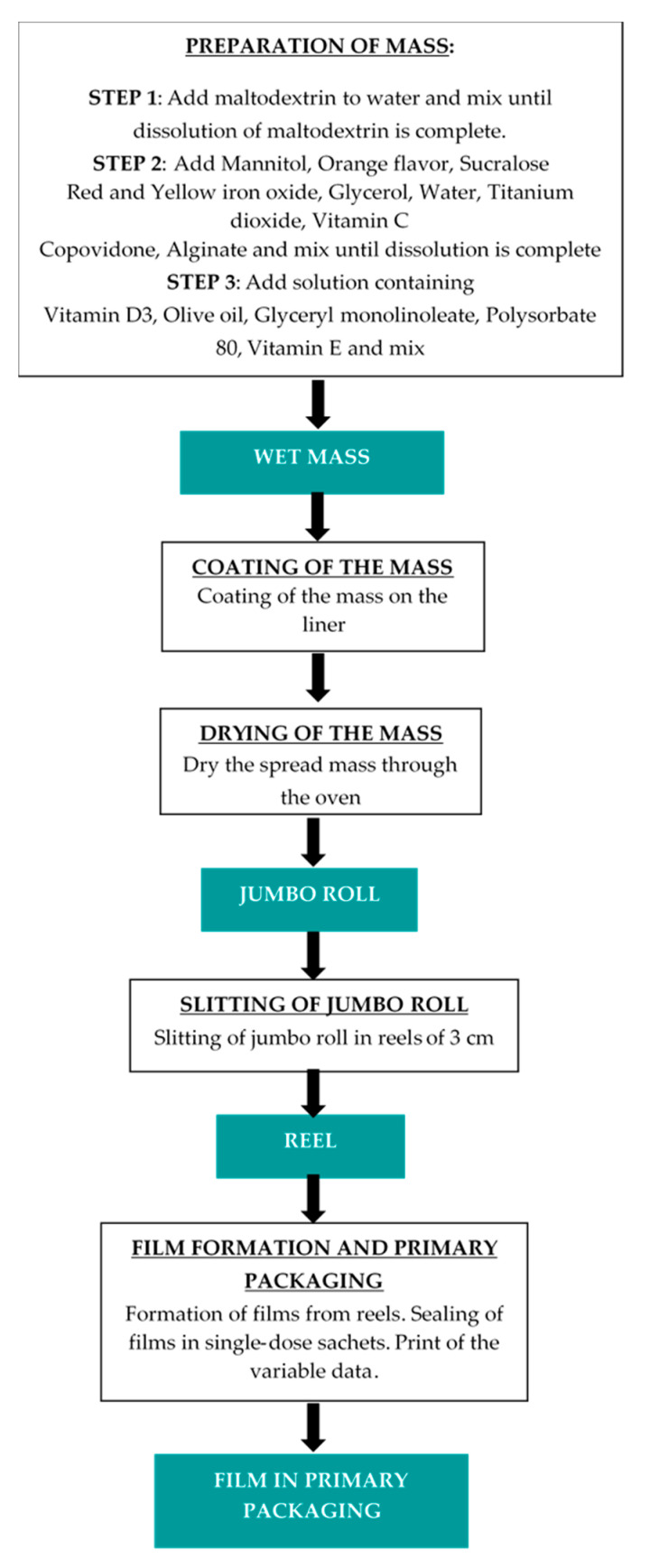
Flow chart of manufacturing steps of vitamin D3 ODF.

The flow chart (Figure 5) briefly describes the steps for the manufacture of orodispersible films containing vitamin D3.

#### 3.2.2. Characterization of Vitamin D3 ODF

##### Aspect, Dimension, and Weight

Orodispersible films were evaluated by visual and organoleptic inspection. The area of films was determined by multiplying length and width of each film and weight of films (*n* = 10) was evaluated.

##### Vitamin D3 Content

The determination of Vitamin D3 assay was carried out by a reversed phase HPLC method.

HPLC method: A 15 μL volume of the sample was injected. A reverse-phase column (KINETEX EVO C18, 150 × 4.6 mm–5 µm) was selected as stationary phase. The mobile phase was prepared mixing 90 volumes of acetonitrile and 10 volumes of water. Flow rate was set at 2.0 mL/min and column temperature at 40 °C. Vitamin D3 was detected at 265 nm. Runtime was set up to 12 min and vitamin D3 retention time was 5 min.

Preparation of Sample solution for assay–2000 IU: Five orodispersible films were accurately weighed and transferred together in a 100 mL amber glass bottle and were dissolved with 10 mL of purified water (exactly measured) stirring for 30 min. 100 mL of MeOH were added with a calibrated pipette, the solution was stirred for 60 min, then filtered with a 0.22 µm PTFE syringe filter and injected in HPLC system.

Each solution was carefully protected from actinic light and air.

##### Disintegration and Dissolution Test

Disintegration test was performed using the Apparatus for disintegration test of tablets (Tecnogalenica, Cernusco sul Naviglio, I), provided with cylindrical discs, described in Ph. Eur. current edition (2.9.1). Dissolution test was performed by a Sotax AT7 Smart dissolution testing unit (Sotax, Aesch, CH), equipped with paddle according to Apparatus 2 of European Pharmacopoeia, current edition (2.9.3). 2000 IU Vitamin D3 orodispersible films were attached to sinkers to avoid floating of the dosage unit. The dissolution medium was 250 mL solution of Triton X-100 0.025% in purified water, maintained at 37.0 ± 0.5 °C. Paddle speed was set up to 50 rpm. 1 mL samples were collected at 5, 15, 30, and 45 min and analyzed by HPLC. Disintegration and dissolution tests were performed on six samples.

##### Loss on Drying

Three orodispersible films were individually weighed and placed in the oven at 105 °C. After 1 h films were taken out of the oven, cooled at room temperature and weighed again. The water content of the films was calculated with the formula

$$\mathrm{Loss}\;\mathrm{on}\;\mathrm{drying}\%\;=\;\frac{weight\;1-\;weight\;2\;}{weight\;1}\times 100$$

where:

*weight 1*: weight of the film before the test (mg)
*weight 2*: weight of the film after the test, in the oven at 105 °C for 1 h (mg)

##### Water Activity

Water activity was determined using Rotronic HW4 instrument (Rotronic Italia, I) on three films.

##### Tensile Properties

Mechanical test was performed using a texture analyzer H5K-T (Tinius Olsen, Horsham, PA, USA) equipped with a 100 N load cell, according to ASTM International Test Method for Thin Plastic Sheeting (D 882-02). Each test strip was longitudinal placed in the tensile grips on the texture analyzer. Initial grip separation was 20 mm and crosshead speed was 50 mm/min. The test was considered concluded at the film break. Tensile strength (TS), elongation at break (EB), and elastic modulus or Young’s modulus (E%) were computed to evaluate the tensile properties of the films.

Tensile strength (TS): was calculated by dividing the maximum load by the original cross-sectional area of the specimen, it was expressed in force per unit area (MPa).

Percent elongation at break (E%): was calculated by dividing the extension at the moment of rupture of the specimen by the initial gage length of the specimen and multiplying by 100.

Elastic modulus or Young’s modulus (Y): was calculated as the slope of the linear portion of the stress strain curve. The results were expressed in force per unit area (Mpa).

Before the test, film thickness of each specimen was measured by using an electronic micrometer (ChemInstruments, Fairfield, OH, USA). Results are the average value of five tests.

##### Microbial Control

The methods to evaluate the total viable aerobic count and the presence of *Escherichia coli* are described in paragraphs 2.6.12 and 2.6.13 of European Pharmacopoeia current edition. The limits of the total count satisfy the requirements of the European Pharmacopoeia current edition, par. 5.1.4 for preparation for oral use. The acceptance criteria are: for total aerobic microbial count (TAMC) ≤10^2^ colony forming units (CFU)/film, for total yeast and molds count (TYMC) ≤10 CFU/film, and absence of *Escherichia coli*.

#### 3.2.3. Stability Study

Stability of 2000 IU Vitamin D3 ODF was studied at long and accelerated term conditions: 25 ± 2 °C and 60% ± 5% relative humidity (RH) and 40 ± 2 °C and 75% ± 5% RH. Each orodispersible film was individually packed in a PET/foil extrusion laminate sachet and it was stored in the climatic chambers in the primary packaging. After three months of vitamin D3 ODFs were evaluated for average weight, vitamin D3 assay, residual water (%), water activity, and microbiology.

#### 3.2.4. Statistical Analysis

The results were expressed as the mean value ± SD. Stability data were analyzed by a *t*-test. A statistical *p*-value of less than 0.05 was considered significant.

## 4. Conclusions

Orodispersible film is an innovative dosage form, currently well investigated at laboratory scale but with few large-scale applications. Most of the existing published literature refers to formulations prepared, for instance, on a petri dish, and are thus almost useless to understand suitability for industrial production. Regarding vitamin D, Zhang et al. [18] has already described an oral fast dissolving film containing vitamin D3 obtained by microemulsifying, but again these films were prepared in laboratory on a bench and no evidence of feasibility of industrial manufacturing was provided.

InTo our knowledge, current work is the first paper reporting industrial preparation of vitamin D in ODF form. An orodispersible film with suitable properties and containing 2000 IU of vitamin D3 was developed and manufactured as innovative dosage form for vitamin D3 supplementation. The food grade ingredients selected for the development of the formula, including the main component maltodextrins, are suitable for manufacturing orodispersible films at industrial scale. Manufacturing process was successfully transferred from lab scale to industrial scale and feasibility of industrial manufacturing for ODF was demonstrated. Residual water content is directly correlated to mechanical properties and a water content in the range 8.0–11.0% *w*/*w* guarantees the suitable mechanical properties of vitamin D3 ODF for production on industrial continuous equipment. Batches of vitamin D3 ODF are chemically and microbiologically stable in the tested period. The selected packaging material guarantees a proper shelf-life of the product and the single pouch is an easy to use packaging. The manufactured films are flexible and can be easily handled, have a pleasant taste and aspect, and are free of organic solvent. The ODFs are homogeneous for vitamin D3 content, disintegrate rapidly in the mouth without water and with no risk of choking, and vitamin D3 is immediately released for quick absorption. Such properties allow good compliance also for specific group populations, such as children and the elderly.

## Figures and Tables

**Table 1:** Composition and excipients function of vitamin D3 orodispersible films.

Ingredients	Function	Composition (%, *w/w*)
Main ingredients:		
Vitamin D3	Active substance	0.06
Maltodextrin	Film polymer	60.45
Glycerol	Plasticizer	12.00
Purified water	Plasticizer	9.00
Mannitol	Plasticizer	7.50
Extra virgin olive oil	Solubilizing agent	3.00
Other ingredients:		
Orange flavor	Flavor	
Copovidone	Bulking agent	
Vitamin C	Antioxidant	
Vitamin E	Antioxidant	
Glycerol monolinoleate	Emulsifier	
Polysorbate 80	Emulsifier	
Titanium dioxide	Opacifier/pigment	
Sucralose	Sweetener	
Yellow iron oxide	Colour	
Red iron oxide	Colour	
Alginate	Thickener	

**Table 2:** Mechanical properties.

Sample	Residual Water (%)	Thickness (μm)	TS (Mpa)	E% (%)	Y (Mpa)
1	8.1	130	5.93	14.0	284
2	8.9	122	4.50	31.9	172
3	8.9	126	5.58	22.0	220
4	8.9	125	4.70	29.9	169
5	9.1	127	4.73	29.4	223
6	9.3	125	4.21	38.1	191
7	9.6	127	2.85	56.0	93
8	9.6	123	2.96	58.7	112
9	10.3	128	2.22	71.7	74
10	10.6	124	1.87	84.4	80

**Table 3:** Stability data of vitamin D3 ODF.

Test		T0 (at the Start of Stability Study)	Three-Month Stability Point	
			25 °C ± 2 °C/ 60% RH ± 5% RH	40 °C ± 2 °C/ 75% RH ± 5% RH
Average weight (mg)		106.1 ± 3.5	106.5 ± 5.6	106.9 ± 2.2
Residual water (%)		9.6 ± 0.3	8.7 ± 0.6	9.4 ± 0.3
Vitamin D3 assay	μg/film	60.3	62.7	61.1
	% of theoretical amount	120.7	125.3	122.1
Water activity		0.538 ± 0.026	0.554 ± 0.017	0.579 ± 0.009
TAMC (CFU/film)		<10 CFU/film	<10 CFU/film	<10 CFU/film
TYMC (CFU/film)		<10 CFU/film	<10 CFU/film	<10 CFU/film
*E. Coli* (unit/film)		Absent/film	Absent/film	Absent/film

## References

[1] Gil A., Diaz J.P., Mesa M.D. (2018). Vitamin D: Classic and novel actions. Ann. Nutr. Metab..

[2] Baeke F., Takiishi T., Korf H., Gysemans C., Mathieu C. (2010). Vitamin D: Modulator of the immune system. Curr. Opin. Pharmacol..

[3] Prietl B., Treiber G., Pieber T.R., Amrein K. (2013). Vitamin D and immune function. Nutrients.

[4] Weir E.K., Thenappan T., Bhargava M., Chen Y. (2020). Does vitamin D deficiency increase the severity of COVID-19?. Clin. Med..

[5] Bergman P., Lindh A.U., Bjorkhem-Bergman L., Lindh J.D. (2013). Vitamin D and Respiratory Tract Infections: A SystematicReview and Meta-Analysis of Randomized ControlledTrials. PLoS ONE.

[6] Nurshad A. (2020). Role of vitamin D in preventing of COVID-19 infection, progression and severity. J. Infect. Public Health.

[7] Amrein K., Scherkl M., Hoffmann M., Sommeregger S.N., Kostenberger M., Berish A.T., Martucci G., Pinz S., Malle O. (2020). Vitamin D deficiency 2.0: An update on the current status worldwide. Eur. J. Clin. Nutr..

[8] Calton E.K., Keane K.N., Newsholme P., Soares M.J. (2015). The Impact of Vitamin D Levels on inflammatory status: A systematic review of immune cell studies. PLoS ONE.

[9] Irfan M., Rabel S., Bukhtar Q., Imran Qadir M.I., Jabeen F., Khan A. (2015). Orally disintegrating films: A modern expansion in drug delivery system. Saudi Pharm. J..

[10] Radicioni M., Castiglioni C., Giori A., Cupone I., Frangione V., Rovati S. (2017). Bioequivalence study of a new sildenafil 100 mg orodispersible film compared to the conventional film-coated 100 mg tablet administered to healthy male volunteers. Drug Des. Dev. Ther..

[11] Scarpa M., Stegemann S., Hsiao W.K., Pichler H., Gaisford S., Bresciani M., Paudel A., Orlu M. (2017). Orodispersible films: Towards drug delivery in special population. Int. J. Pharm..

[12] Wasilewska K., Winnicka K. (2019). How to assess orodispersible film quality? A review of applied methods and their modifications. Acta Pharm..

[13] Cilurzo F., Montanari L., Minghetti P. (2004). Self Supporting Film for Pharmaceutical and Food Use. Patent.

[14] Cilurzo F., Di Grigoli M., Minghetti P., Pagani S. (2014). Orodispersible Films Having Quick Dissolution Times for Therapeutic and Food Use. Patent.

[15] Thabet Y., Breitkreutz J. (2018). Orodispersible films: Product transfer from lab-scale to continuous manufacturing. Int. J. Pharm..

[16] Cuomo F., Cinelli G., Chirascu C., Marconi E., Lopez F. (2020). Antioxidant effect of vitamins in olive oil emulsion. Colloids Interfaces.

[17] Im S., Nam T.G.N., Lee S.G., Kim Y.J., Chun O.K., Kim D.O. (2014). Additive Antioxidant Capacity of Vitamin C and Tocopherols in combination. Food Sci. Biotecnol..

[18] Zhang M., Zhang T., Zou Y., Han P., Liu K. (2019). Self-microemulsifying oral fast dissolving films of vitamin D3 for infants: Preparation and characterization. Food Sci. Nutr..

